# Hermes (Rbpms) is a Critical Component of RNP Complexes that Sequester Germline RNAs during Oogenesis

**DOI:** 10.3390/jdb4010002

**Published:** 2016-01-19

**Authors:** Tristan Aguero, Yi Zhou, Malgorzata Kloc, Patrick Chang, Evelyn Houliston, Mary Lou King

**Affiliations:** 1 Department of Cell Biology, University of Miami School of Medicine, Miami, FL 33136, USA; t.aguero@med.miami.edu (T.A.); yzhou1999@126.com (Y.Z.); 2 Houston Methodist Research Institute, The Houston Methodist Hospital, Bertner Avenue, Houston, TX 77030, USA; mkloc@houstonmethodist.org (M.K.); 3 Laboratoire de Biologie du Développement de Villefranche-sur-mer (LBDV), Sorbonne Universités, UPMC Univ Paris 06, CNRS, Villefranche-sur-mer 06230, France; chang@obs-vlfr.fr (P.C.); houliston@obs-vlfr.fr (E.H.)

**Keywords:** *Xenopus* oogenesis, nanos1 RNA localization, germline, germinal granules, RNP particle, *nanos* ribonucleoprotein complex

## Abstract

The germ cell lineage in *Xenopus* is specified by the inheritance of germ plasm that assembles within the mitochondrial cloud or Balbiani body in stage I oocytes. Specific RNAs, such as *nanos1*, localize to the germ plasm. *nanos1* has the essential germline function of blocking somatic gene expression and thus preventing Primordial Germ Cell (PGC) loss and sterility. Hermes/Rbpms protein and *nanos* RNA co-localize within germinal granules, diagnostic electron dense particles found within the germ plasm. Previous work indicates that *nanos* accumulates within the germ plasm through a diffusion/entrapment mechanism. Here we show that Hermes/Rbpms interacts with *nanos* through sequence specific RNA localization signals found in the *nanos*-3′UTR. Importantly, Hermes/Rbpms specifically binds *nanos*, but not *Vg1* RNA in the nucleus of stage I oocytes. *In vitro* binding data show that Hermes/Rbpms requires additional factors that are present in stage I oocytes in order to bind *nanos1*. One such factor may be hnRNP I, identified in a yeast-2-hybrid screen as directly interacting with Hermes/Rbpms. We suggest that Hermes/Rbpms functions as part of a RNP complex in the nucleus that facilitates selection of germline RNAs for germ plasm localization. We propose that Hermes/Rbpms is required for *nanos* RNA to form within the germinal granules and in this way, participates in the germline specific translational repression and sequestration of *nanos RNA*.

## 1. Introduction

Localization of specific RNAs to subcellular domains is one mechanism by which cells restrict protein synthesis in time and space. During *Xenopus* oogenesis, selected RNAs are localized and retained within the vegetal cortex at two distinct time periods. Most RNAs essential to forming the germline localize very early in oogenesis within a macroscopic structure called the mitochondrial cloud (MC) or Balbiani body [1,2,3,4,5,6,7,8]. There, the germ plasm assembles and contains all the components, including germinal granules, required and sufficient to determine germ cell identity [9,10,11,12]. One known component of germinal granules is *nanos* RNA whose product is essential to the preservation of the germline in many diverse species including *Drosophila*, *Xenopus*, and mouse [3,13,14,15,16,17,18].

Another set of RNAs, previously distributed throughout stage I oocytes, is actively localized via a microtubule kinesin-dependent mechanism at later oocyte stages [19,20,21]. RNAs following this so-called late pathway include the transcription factor *VegT* and a *TGFβ* family member *Vg1*. The products of these genes are required for specifying and patterning the endoderm and mesoderm [22,23,24,25]. Why *Vg1* remains uniformly distributed in the cytoplasm while *nanos* RNA accumulates in the MC remains an unanswered question. The selection process for the different localization pathways is not well understood but is essential for the creation of the future germline and primary germ layers.

Time-lapse Confocal microscopy and FRAP (Fluorescence Recovery After Photobleaching) analysis show that injected fluorescently labeled *nanos* RNA form particles that disperse evenly throughout the ooplasm in stage I oocytes. Over time, these particles became progressively immobilized, but only within the MC where they form larger aggregates reminiscent of germinal granule formation [6]. Identification of the cis- and trans-acting factors involved in the selection process for either the early or late localization pathway is certainly an important step towards a full mechanistic understanding of RNA localization. Although there are exceptions [26], virtually all localization signals (LS) reside in the 3′UTR, consist of multiple elements, and display considerable functional redundancy [27,28]. Clustering of these repeated elements may be critical to facilitating interactions between different proteins in the localization machinery [29,30,31].

*Vg1* and *VegT* are directed to the vegetal pole by a 340-nt localization signal (LS) in their 3′UTR. *In vitro* and *in vivo* UV crosslinking analyses reveal six proteins that interact directly with both the *Vg1* and *VegT*-LS [30,31,32]. Within this signal are certain small repeated elements called E2 and VM1 that are required for localization [33,34,35,36,37,38] and have been found to function as binding sites for the specific RNA-binding proteins Vg1RBP/Vera and hnRNP I, respectively. *Vg1*-LS mutants in E2 and VM1 sites fail to bind their respective proteins *in vitro* and fail to localize *in vivo* [33,35,38,39,40]. Two other proteins Prrp and Xstau also bind *Vg1* RNA and co-localize with it at the vegetal cortex [21,41,42]. RNA localization begins as a recognition event, most likely in the nucleus, and has been linked to splicing events [42,43,44]. More recently, Vg1RBP/Vera and hnRNP I were found to bind to each other and to *Vg1* in the nucleus while Prrp and Xstau were recruited to the RNP complex only in the cytoplasm [42]. These findings strongly suggest that RNA binding to distinct proteins in the nucleus segregates the early and late pathways.

The *nanos*-3′UTR has two different localization signals. The 240 nt mitochondrial cloud localization signal (MCLS) is both required and sufficient to direct *nanos* into the MC [4,6]. In addition, the 160 nt germinal granule localization element (GGLE) is required to direct *nanos* into germinal granules, an event that requires the prior functioning of the MCLS [45]. The *nanos* MCLS was shown to bind directly to Vg1RBP/Vera and hnRNP I *in vitro*, consistent with its ability to use the late pathway after injections into late staged oocytes [29,46]. However, endogenous Vg1RBP/Vera appears excluded from the MC as shown by immunolocalization [6]. Furthermore, the ER-*nanos* association/entrapment event does not involve Vg1RBP/Vera, a protein implicated in linking *Vg1* RNA to the ER [33]. How then can the early and late pathways be distinguished and sorted into different cellular domains? Clearly, proteins that bind early pathway RNAs like *nanos*, but not *Vg1* RNA must exist in the stage I oocyte.

The nature of the RNA-protein interactions operating in the early pathway that mediate the steps of RNA selection, entrapment, and translational regulation remain unknown. Complicating our understanding of these processes are RNAs such as *hermes/rbpms* that localize using both early and late pathways [25]. Here we describe work on the RNA binding protein Hermes/Rbpms. Hermes/Rbpms is an RNA Recognition Motif (RRM) family member originally found to play a role in embryonic heart development [47] and later re-discovered in a screen for vegetally localized maternal RNAs [25]. Functional studies have linked it with myocardial differentiation [48] and cell division within the vegetal hemisphere [25], but its mode of operation in these events remains unclear although a negative role in translation has been proposed [49].

We find that Hermes/Rbpms protein is present in the MC, throughout the cytoplasm and in the nucleus of stage I oocytes. However, unlike other RNA-binding proteins, we confirm that Hermes/Rbpms co-localizes with *nanos* in the MC, concentrating in the germ plasm forming region and within the germinal granules [49]. Importantly, we show that Hermes/Rbpms protein forms distinct particles and associates with *nanos* but not *Vg1* or *VegT* RNA within the nucleus. Hermes/Rbpms binding to nanos 3′UTR requires an unknown component present in stage I but not stage VI oocyte extracts. The UGCAC repeats essential for *nanos* RNA MC localization are also required for Hermes/Rbpms binding to the MCLS. In addition, Hermes/Rbpms binds the GGLE domain, a region that does not contain UGCAC elements but does contain two VM1 hnRNP I binding sites. Further, we find that the terminal 34 amino acids in Hermes/Rbpms, a region conserved with human RBPMS, is required to form homodimers as well as to bind the *nanos* 3′UTR.

Taken together, our observations show that Hermes/Rbpms is an important component of the *nanos* RNP particle. Hermes/Rbpms functions as a homodimer in concert with other proteins to facilitate granule formation. We propose that Hermes/Rbpms binds *nanos* RNA in the nucleus in association with hnRNP I, initiating a series of events that results in *nanos* but not *Vg1* entering the early pathway. We suggest that, by incorporating *nanos* RNA into a Hermes/Rbpms nuclear particle, *nanos* RNA is effectively prevented from being translated after it exits the nucleus. To our knowledge, this is the first evidence for a nuclear RNA-binding protein specific for the early pathway.

## 2. Experimental Section

### 2.1. Oocytes and Microinjection

Stage I/II oocytes were obtained after collagenase treatment of surgically removed ovarian tissue as previously described [6]. Myc-Hermes/Rbpms mRNA was transcribed with mMessage mMachine (Ambion) and 300 pg injected into stage I/II oocytes. Injected oocytes were cultured for two days in oocyte culture medium (OCM: 60% Liebovitz L-15 (Gibco), 0.04% bovine serum albumin, 1 mM l-glutamine, and 5 µg/mL gentamycin).

### 2.2. Myc-Hermes/Rbpms Immunoprecipitation

After the two-day culture period, the nuclear and cytoplasmic fractions were manually isolated in OR2 buffer. After injection of 1nl of water, the nucleus emerged from the needle-puncture made in the plasma membrane. Whole oocytes, ooplasms, and nuclear samples were homogenized in YSS buffer (50 mM Tris pH 8.0, 50 mM NaCl, 0.1% NP40, 0.1U/µL superRNAsin, 1X protease inhibitors from Roche, 0.5 mM DTT, 100 mM sucrose) and centrifuged at 16,000 g for 3 min to remove the insoluble materials. Anti-myc antibody (9E10, Affymetrix) and protein G beads were added to the supernatant. The mixture was incubated at 4 °C overnight. The beads were then washed with YSS buffer four times. The bound RNAs were recovered with proteinase K digestion and analyzed by RT-PCR using the primers: Nanos-F, gaggctacacttgccctttg and Nanos-R, gcccattagtggtgcagaat; Vg1-F, atgcctattgcttctatttgc and Vg1-R, ggtttacgatggtttcactca and VegT-F, caagtaaatgtgagaaaccg and VegT-R, caaatacacacacatttccc). Possible cross contamination between ooplasm and nuclei during isolation procedures was monitored by western blotting with anti-alpha tubulin (12G10; 0.5 µg/mL) and anti-nucleoplasmin (B7-1A9; 1.5 µg/mL) antibodies respectively (Developmental Studies Hybridoma Bank).

### 2.3. Mutagenesis, Cloning, and RNA Synthesis

Hermes/Rbpms deletion mutants were constructed as follows:

Hermes/Rbpms deletion A (a.a. 101–121 were deleted): the N terminal fragment was amplified with Hermes/Rbpms ATTTAGGTGACACTATAGAgtacaccatgagcggcatcaagtcagaca (forward) and Hermes/Rbpms-DA1 (CTTTGTGTTGGCCTTTGC). The C terminal fragment was amplified with Hermes/Rbpms-DA2 (GCAAAGGCCAACACAAAGCACTTCATTGCACGAGAT) and Hermes/Rbpms TTTTTTTTTTTTTTTTTAACAAAACTGCCGAGACT (reverse 1). The two fragments were then fused by PCR. Hermes/Rbpms deletion B (a.a. 122–161 were deleted): the N terminal fragment was amplified with Hermes/Rbpms ATTTAGGTGACACTATAGAgtacaccatgagcggcatcaagtcagaca (forward) and Hermes/Rbpms-DB1 (TGCGCCAAGTGCTGGGTG). The C terminal fragment was amplified with Hermes/Rbpms-DB2 (CACCCAGCACTTGGCGCAGCTTTCACATACCCTGCT) and Hermes/Rbpms TTTTTTTTTTTTTTTTTAACAAAACTGCCGAGACT (reverse 1). The two fragments were then fused by PCR. Hermes/Rbpms deletion C (a.a. 164–198 were deleted) was amplified with Hermes/Rbpms ATTTAGGTGACACTATAGAgtacaccatgagcggcatcaagtcagaca (forward) and Hermes/Rbpms TTTTTTTTTTTTTTTTTAAGCAGCATGTGGAATGGC (reverse 2). The resulting PCR products from Hermes/Rbpms deletion mutants were used as templates for *in vitro* transcription (mMessage mMachine, Ambion, Austin, TX, USA). The RNAs were translated in reticulocyte lysates following the manufacturer’s instructions (Promega).

Myc-Hermes/Rbpms: plasmid pCS2+6Xmyc Hermes/Rbpms was designed as detailed in Song *et al.*, 2007 [49]. hnRNP I: plasmid PSP64TSN-RLMCS-p60-Flag was a gift from Dr. K. Mowry (Brown University). Myc-Nanos: Nanos coding region was amplified with MT-nanos cagcttgaattca ATGGATGGCGGTCTCTGC [49] (forward) and MT-nanos actagtctcgagTCAGTGTCTCAGCTTTGG (reverse). The PCR product was digested with EcoRI and XhoI and cloned into the pCS2+MT vector (from D. Turner).

GST-Hermes/Rbpms-C: The Hermes/Rbpms C terminal region was amplified with GST-Hermes/Rbpms-C ACTGACGAATTCATGGCC AAGAACAAACTA (forward) and GST-Hermes/Rbpms (AGCTATCTCGAGTTAACAAAACTGCCGAGA (reverse). The PCR product was digested with EcoRI and XhoI and cloned into pGEX-5x-1.

Sense RNAs were transcribed from NotI-linearized plasmids with SP6 RNA polymerase and the mMessage mMachine kit (Ambion, Austin, TX). For fluorescent probes, 600 µM Alexa 488-UTP or tetramethylrhodamine-UTP (Molecular Probes, Eugene, OR, USA) was used exactly as described in Chang *et al.*, 2004 [6]. Alexa 488-labeled *nanos*-3UTR was injected 20 h before fixation and antibody staining.

### 2.4. In Vitro Translation

^35^S-Methionine labeled Hermes proteins were synthesized in rabbit reticulocyte lysates (Promega) using capped *in vitro* transcribed RNA following the manufacturer’s protocol. For radiolabeling, 20 µCi of ^35^S-methionine was included (New England Nuclear, Boston, MA, USA, NEG009A, 1200 Ci/mmol). Samples were immunoprecipitated under denaturing conditions with anti-myc antibody and analyzed by SDS-PAGE. The bound protein was quantitated using a PhosphorImager™ (Storm 840, Molecular Dynamics/GE Healthcare Life Sciences, Pittsburgh, PA, USA) with the ImageQuant software package (8.1, GE Healthcare Life Sciences, Pittsburgh, PA, USA).

### 2.5. RNA-AADA Pull-Down

RNA was transcribed with mMessage Machine (Ambion) and purified by LiCl precipitation. 0.2–0.5 µg of RNA was oxidized with 1 mg/mL of sodium periodate in 0.1 M NaAc (pH 5.0) at room temperature for one hour. The oxidized RNA was then immobilized on adipic acid dehydrazide agarose (AADA) beads (Sigma, St. Louis, MO, USA) in 0.1 M NaAc (pH 5.0) at 4 °C over night. The RNA-AADA beads were washed with 1x TPB buffer (60 mM Tris pH7.4, 10 mM MgCl2, 80 mM NaCl, 0.1% Triton X-100, 10% glycerol).

Stage I/II oocytes were homogenized in the presence of 1x protease inhibitor (Roche), DTT (0.3 mM) and heparin (5 mg/mL). Insoluble materials were removed by centrifugation at 16,000 g for 3 min. The supernatant was mixed with *in vitro* translated Myc-Hermes/Rbpms labeled with ^35^S-methionine for 5 min at room temperature. The mixture was then precleared for 30 min with AADA agarose beads prewashed with 1x TPB buffer at room temperature. After centrifugation at 16,000 g for 3 min, the supernatant was mixed with RNA-AADA beads at room temperature for 5 min. The beads were then washed three times with 1x TPB buffer. The radio-labeled bound proteins were analyzed by SDS-PAGE and visualized using a PhosphorImager™.

### 2.6. Yeast-Two-Hybrid

We identified proteins interacting with Hermes/Rbpms using the Two-Hybrid System 3 from Clontech following their detailed protocol (Clontech Yeast Protocol Handbook, Matchmaker 3 manual, 2 Hybrid System TRAFO Protocol). System 3 uses yeast strain AH109, which includes four reporter genes (ADE2, HIS3, lacZ, and MEL1) to help reduce false positives. High stringency conditions were used plating yeast on SD/-Ade/-His/-Leu/-Trp/X-a-gal plus 10mM 3-AT following the procedures presented by Gietz and Schiestl [50]. A *Xenopus* oocyte two-hybrid cDNA library cloned in pGAD10 was obtained from Clontech. Two hundred micrograms of plasmid DNA from each library was transformed into competent yeast L40-coat/WT cells as described by Clontech. The Hermes/Rbpms coding region was used as bait and amplified with pAS1-Hermes/Rbpms AGCTAGGCCATGG AGagcggcatcaagtcagac (forward) and pAS1-Hermes/Rbpms AGTCAGGTCG ACTTAACAAAACTGCCGAGA (reverse). The PCR product was cloned into the pAS1 vector (from Dr. S. Elledge) using NcoI and SalI. Hermes/Rbpms was fused to the C terminus of Gal4 DNA binding domain by PCR.

### 2.7. Immunofluorescence Microscopy and Antibodies

Oocytes were fixed and processed exactly as described in Chang *et al.*, 2004 [6] for immunofluorescence microscopy. Anti-Hermes/Rbpms antibody was generated against a nonconserved peptide region (AHFIARDPYDLTGAA) and purified as described in Zearfoss *et al.*, 2004 [25]. Anti- Vg1RBP/Vera antibodies from Nancy Standart (University of Cambridge, Oxford, UK), anti-alpha-tubulin mouse mAb DMIA (Sigma), used at 1:1000, and preabsorbed rhodamine- or fluorescein isothiocyanate-labeled anti-Ig antibodies (Jackson Immunoresearch Laboratories, West Grove, PA, USA), used at 1:75 to 1:150. Anti-hnRNP I antibodies were a gift from K. Mowry (Brown University, RI, USA) used at 1:100 and secondary antibody was either FITC-Goat anti-rabbit IgG 1/100 or Alexa^®^ 568-conjugated goat anti–rabbit (ThermoFisher Scientific , NY, USA) at 1:100 [42]. Rat monoclonal anti-GRP94 1/250 and Rhodamine-anti-rat 1/75 was used as an ER marker as described in Beckhelling *et al.*, 2003 [51] (StressGen Biotechnologies, Victoria, BC, Canada). Imaging on the Leica SP2 confocal.

### 2.8. Ultrastructural Analysis

All hybridization and post-hybridization steps, embedding, and sectioning were done as described in Kloc *et al.* 2001 [10]. *Nanos* (Xcat2) RNA and Hermes/Rbpms protein were identified by *in situ* hybridization as described in Song *et al.*, 2007 [49] and Kloc *et al.*, 2002 [3].

### 2.9. Ethical Statements

The animal protocols used in this work were evaluated and approved by the Institutional Animal Care and Use Committee (IACUC) of the University of Miami. All activities are in compliance with federal, state, and institutional regulations. The University was granted full accreditation by the Association for Assessment and Accreditation of Laboratory Animal Care, International (AAALAC) in February 2005 and received its current re-accreditation in 22 October 2013. In addition, University of Miami is licensed by the U.S. Department of Agriculture (USDA) and has filed a Letter of Assurance with the Office of Laboratory Animal Welfare (OLAW), U.S. Department of Health and Human Services (DHHS).

## 3. Results

### 3.1. Hermes/Rbpms Co-Localizes with Nanos RNA within Germinal Granules

**Figure 1:**
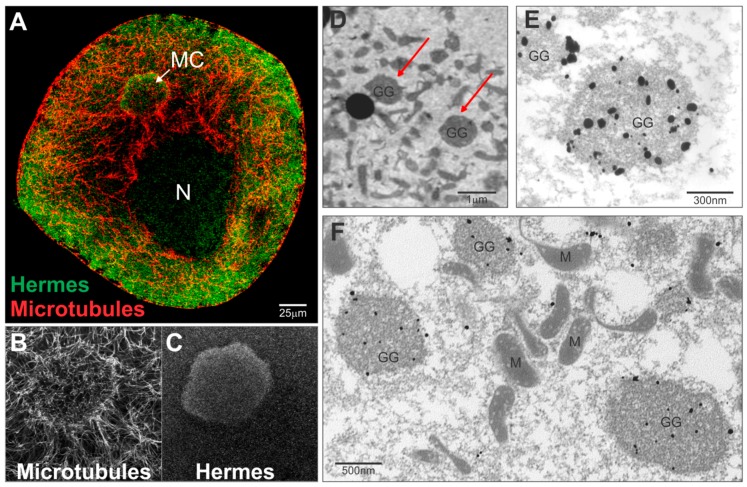
Hermes/Rbpms protein and *nanos* RNA localize within germinal granules unique to germ plasm. Stage I oocyte. (**A**) Immunofluorescence (IF) and confocal microscopy showing Hermes/Rbpms protein (**green**) is ubiquitous in stage I oocyte including the mitochondrial cloud (MC), nucleus (N) and ooplasm; microtubules (**red**). Enlarged image from (**A**) showing microtubule organization around MC (**B**) and Hermes/Rbpms (**C**); Note how Hermes/Rbpms is enriched within the germ plasm region of the MC; (**D**) Electron micrograph showing a region of germ plasm within the MC of stage I oocyte. Red arrows indicate two germinal granules; (**E**) *Nanos* RNA is localized within germinal granules in stage I oocytes as is Hermes/Rbpms protein (**F**). Electron microscopy *in situ* hybridization with antisense Digoxigenin labeled *nanos* RNA was visualized with nanogold conjugated anti-Dig antibody and silver enhancement. Electron microscopy immunostaining with Hermes/Rbpms polyclonal antibody [49], anti-rabbit nanogold conjugated secondary antibody and silver enhancement. Scale bars are as indicated. GG (germinal granules); M (mitochondria); N (nucleus).

In agreement with Zearfoss *et al.*, 2004 [25], we found that Hermes/Rbpms is present within the mitochondrial cloud (MC) and throughout the cytoplasm of stage I oocytes. However, we also found Hermes/Rbpms protein in the nucleus (Figure 1). Hermes/Rbpms RNA follows the same cellular expression pattern as its protein and suggests that Hermes/Rbpms may have several roles during oogenesis depending on which RNA it may bind [49,52]. Previous work showed that Hermes/Rbpms protein over-expressed at the vegetal, but not the animal pole, formed large aggregates that contain RNA [47,48,53]. We also confirmed by ultrastructural immune-localization that within the MC, Hermes/Rbpms and *nanos* RNA are predominantly localized within the germinal granules (Figure 1; [49]). These observations led us to ask if Hermes/Rbpms protein and *nanos* RNA directly interact with each other.

### 3.2. Hermes/Rbpms Protein Specifically Binds the Nanos Mitochondrial Cloud Localization Signal

**Figure 2:**
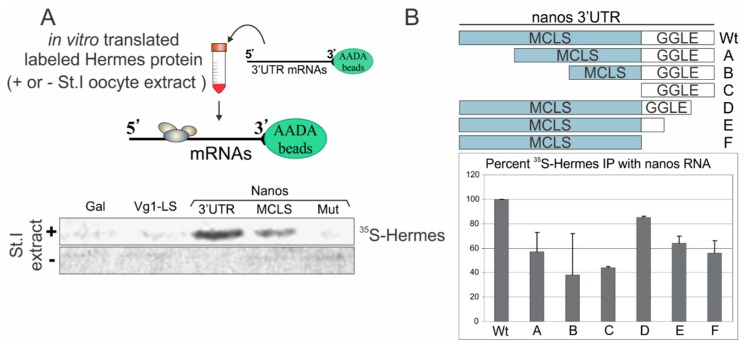
Hermes/Rbpms specifically associates with *nanos* RNA in a UGCAC dependent manner. (**A**) Hermes/Rbpms interacts with *nanos* but not *Vg1* RNA. *Vg1* 3′UTR Localization Experimental design is shown at top. *Vg1* Localization Signal (LS), *nanos1* 3′UTR, *nanos* Mitochondrial Cloud Localization Signal (MCLS), and a *nanos* mutant MCLS (mut) were tested for Hermes/Rbpms binding. Individual RNAs were immobilized on AADA agarose beads and mixed with *in vitro* translated Hermes/Rbpms protein labeled with ^35^S-methionine. Bound proteins were analyzed by SDS-PAGE and autoradiography. *Galactosidase* (Gal) RNA served as a negative control. Hermes/Rbpms only bound the *nanos1-3*′*UTR* and only when reactions included stage I/II oocyte extracts and not reticulocyte lysates alone. Note: the 3′UTR binds more Hermes/Rbpms than the MCLS alone suggesting the GGLE may also bind Hermes/Rbpms; (**B**) Both the MCLS and GGLE are required for wild-type levels of Hermes/Rbpms binding and require stage I oocyte extract. The deletion mutants diagramed were immobilized on AADA beads and analyzed as described in Methods. Error bars indicate standard deviation of three experiments. Hermes/Rbpms binding to the full-length *nanos* 3′UTR is set at 100%. Note: both regions contribute to Hermes binding.

To address whether Hermes/Rbpms specifically binds *nanos* RNA, the *nanos* 3′UTR was chemically cross-linked to AADA beads and the beads incubated with reticulocyte lysate containing *in vitro* translated radio-labeled Myc-Hermes/Rbpms protein [54,55]. Bound protein was analyzed by SDS-PAGE and visualized by autoradiography. Myc-Hermes/Rbpms was pulled down with the *nanos* 3′UTR, but only when the lysate was supplemented 1:1 with stage I oocyte extract (+). These findings strongly suggested another protein(s) is required for Hermes/Rbpms to efficiently associate with the *nanos* 3′UTR [48]. Alternatively, Hermes/Rbpms may need to be modified in some way before being competent to bind *nanos*. Importantly, Hermes/Rbpms did not bind the *Vg1* localization signal (LS), suggesting a level of discrimination between these two RNAs at the level of Hermes/Rbpms protein binding (Figure 2). Galactosidase (Gal) served as a negative control and showed little binding capability.

Within the 240 nt MCLS are six UGCAC motifs previously shown to be essential for *nanos* RNA to localize within the MC. The UGCAC sequences likely mediate redundant protein binding to the signal [6,28,29,56]. In the pull-down assay, Hermes/Rbpms consistently bound the *nanos* 240 nt-MCLS, but with reduced affinity compared to the full length 3′UTR. To test the importance of the MCLS UGCAC repeats for Hermes/Rbpms binding, we introduced substitution mutations in all six, altering them to non-cognate sequences UUGGG or AGGCC. Previous studies with Par-Clip indicate CAC sites are preferred for Hermes/Rbpms binding [52]. But we found that simply changing the UGCAC repeats to UUCAC prevented Hermes/Rbpms binding to the 240 nt-MCLS and significantly impaired MC entrapment [6]. Hermes/Rbpms failed to bind to the *nanos* 240 nt-MCLS lacking the UGCAC repeats in the pull-down assay (Mut in Figure 2). Thus, the UGCAC sites required for localization are also required for Hermes/Rbpms to bind the *nanos* MCLS. These results show that Hermes/Rbpms binds *nanos* RNA specifically in a UGCAC dependent manner and loss of Hermes/Rbpms binding is correlated with loss of *nanos* RNA localization into the MC. However, the results do not address whether Hermes/Rbpms directly interacts with the UGCAC repeats.

The 160 nt germinal granule localization element (GGLE) just downstream of the MCLS directs *nanos* into germinal granules, an event that requires the prior functioning of the MCLS [45]. The reduced binding of Hermes/Rbpms to the 240 nt-MCLS compared to the entire 3′UTR raised the possibility that Hermes/Rbpms also associated with the GGLE. To determine if Hermes/Rbpms could bind the GGLE alone, deletion mutants were introduced into the 3′UTR by PCR. All deletions showed a decreased affinity to Hermes/Rbpms, indicating both the MCLS and GGLE are required for optimal binding (Figure 2B) The GGLE alone retained 40% binding activity compared to 58% by the MCLS. The GGLE region does not contain any UGCAC sites but several VM1 sites. Thus both regions of the *nanos* 3′UTR contribute to Hermes/Rbpms binding (Figure 2B; compare lanes C and F with WT). These results indicate that Hermes/Rbpms can associate with the *nanos* GGLC in the absence of UGCAC sites or the MCLS, suggesting a role in germinal granule formation.

### 3.3. Mapping Hermes/Rbpms for Regions Required for Nanos-Hermes/Rbpms Interaction

**Figure 3:**
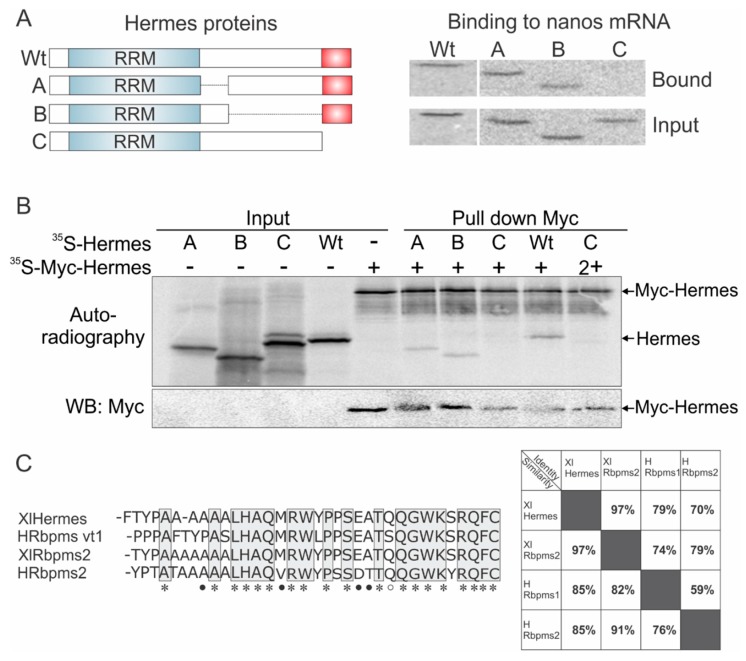
The carboxyl terminal 34 amino acids of Hermes/Rbpms are required for *nanos1* binding and for Hermes/Rbpms dimerization. (**A**) Three deletion mutants of Hermes/Rbpms are diagramed showing the RRM domain and the hydrophilic carboxyl terminal 34 amino acids (**red** box). ^35^S-methionine labeled Hermes/Rbpms and its deletion mutants were analyzed for their ability to be pulled-down by *nanos1* 3′UTR immobilized on AADA beads. Wild-type and mutant protein (input) used in reaction and bound are shown by SDS-PAGE. Note that only the last 34 amino acids were required for *nanos* binding in the presence of the Hermes/Rbpms RRM; (**B**) The presence of Hermes/Rbpms homodimers was detected by autoradiography as two bands. Note that Hermes/Rbpms missing the terminal 34 amino acids failed to form a dimer. As *nanos* RNA was not present, Hermes/Rbpms formed homodimers in the absence of RNA; (**C**) C-terminal region involved in Hermes/Rbpms homodimerization and binding to the *nanos* 3'UTR. Alignment of conserved terminal 34 amino acids (AA) of Human and *Xenopus* Hermes/Rbpms proteins. An * (asterisk) indicates a fully conserved AA. Black dots indicate conservation between groups of strongly similar properties (scoring > 0.5 in the Gonnet PAM 250 matrix). A white dot indicates weakly similar properties (scoring ≤ 0.5 in the Gonnet PAM 250 matrix), Clustal Omega, EMBL-EBI, UK. Table shows the high level of identity and similarity between frog and human Hermes/Rbpms proteins.

Proteins involved in RNA localization often function as dimers or oligomers, operating as partners with other proteins to confer specificity to the RNA recognition event [15,57]. Previous work has shown that Hermes/Rbpms forms homodimers and can oligomerize [48]. Therefore, we asked what region of Hermes/Rbpms was required for its interaction with *nanos* RNA and whether that region was required for Hermes/Rbpms to form homodimers. Hermes/Rbpms protein has an N terminal RRM domain required but not sufficient for binding polyA+ RNAs [48]. Keeping the RRM domain intact, we introduced three deletion mutations within the C-terminus and determined what region was required for *nanos* binding. Each deletion mutant as well as wild-type Hermes/Rbpms was first translated *in vitro* and shown to translate equally well by western blotting (Figure 3A). Each myc-tagged Hermes/Rbpms protein was then tested as previously described for the ability to bind *nanos* RNA immobilized on AADA beads in the presence of stage I oocyte extract. The results showed that only the terminal 34 amino acids were absolutely required for *nanos* binding in the presence of the RRM (Figure 3A, mutant C). Therefore, the Hermes/Rbpms RRM domain was not sufficient for nanos RNA interaction, but binding also required the very hydrophilic C-terminus of Hermes/Rbpms (Figure 3C).

To determine what region of Hermes/Rbpms is required for homodimerization, Hermes/Rbpms wild-type and mutant proteins, both with or without myc tags, were translated *in vitro* in the presence of ^35^S-methionine. Each synthesized radiolabelled Hermes/Rbpms protein was mixed with its myc-tagged counterpart and incubated prior to immunoprecipitation (Figure 3B). After immunoprecipitation with anti-Myc antibody, the co-precipitates were analyzed by western blotting and autoradiography (PhosphorImager). Myc-Hermes/Rbpms migrates at 38 kDa while wild-type Hermes/Rbpms at 28 kDa. Homodimers between myc-Hermes/Rbpms and Hermes/Rbpms are easily detected as two bands. Only mutant C, missing the last 34 amino acids, failed to form a homodimer (Figure 3B). Taken together, the results indicate that the same 34 amino acids are required for Hermes/Rbpms to form homodimers and to be competent to associate with *nanos* RNA. Interestingly, the last 34 amino acids are 85% conserved between *Xenopus* and human Hermes/Rbpms (Figure 3C).

### 3.4. Hermes/Rbpms Interacts with hnRNP I

**Figure 4:**
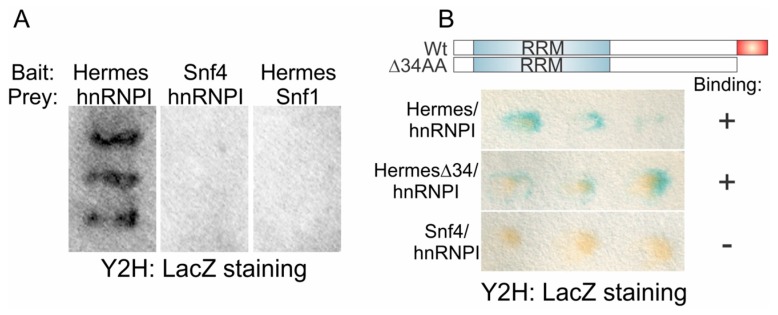
Hermes/Rbpms interacts with another Nanos1 binding protein: To identify proteins that might interact with Hermes/Rbpms during early oogenesis, a *Xenopus* cDNA library (Clontech) was screened using a yeast two-hybrid (Y2H) system. (**A**) Full length Hermes/Rbpms fused with the DNA binding domain (BD) of the transcription factor Gal4 served as bait. Candidate interacting proteins were fused to the Gal4-activation domain (AD) and served as prey. The reporter gene B-galactosidase, driven by the Gal4 binding site, was positively transcribed as the result of Hermes/Rbpms and hnRNP I (heterogeneous nuclear ribonucleoprotein I) interaction. Two proteins involved in yeast glucose metabolism, Sfn4 and Sfn1, were used as controls to discard false positive results. Snf4/Gal4-BD was used as a bait and Snf1/Gal4-AD as a prey to check false interactions with hnRNP I and Hermes/Rbpms protein respectively; (**B**) The C-terminal 34 amino acids (in red) required for Hermes/Rbpms to dimerize and bind *nanos* RNA are not required to interact with hnRNP I. Hermes/Rbpms protein lacking the terminal 34 AA was used as bait and hnRNP I as prey. Induction of B-galactosidase indicated a positive interaction between bait and prey.

Hermes/Rbpms binding to *nanos* required the addition of oocyte extract to the assay (Figure 2A) suggesting the presence of a co-factor that facilitates Hermes/Rbpms binding. Alternatively, or in addition, an oocyte factor may promote a post-translational modification required for *nanos* association. We favor the former explanation, as endogenous and translated Hermes/Rbpms appeared to have the same molecular mass after gel fractionation. To identify oocyte proteins capable of interacting with Hermes/Rbpms, we screened an expression library (Clontech) made from *Xenopus* oocyte mRNA containing 2.5 × 10^6^ independent clones with an average insert size of 1.5 kb in a yeast-two-hybrid assay. The known RNA-binding protein hnRNP I was identified from approximately 700 × 10^3^ clones (Figure 4). Previous work had shown that hnRNP I is present in early staged oocytes and has a role in *Vg1* RNA localization [39]. hnRNP I seemed a strong candidate and was selected for further analysis. We asked if the Hermes/Rbpms:hnRNP I interaction in the yeast-2-hybrid assay required the last 34 amino acids (ΔC162-196) of Hermes/Rbpms. A similar failure in binding of hnRNP I would suggest this protein is the missing factor found in oocyte extracts required for Hermes/Rbpms binding to *nanos.* However, mutC-Hermes/Rbpms still interacted efficiently with hnRNP I while SNF4, the negative control, did not (Figure 4). Therefore, Hermes/Rbpms-hnRNP I interaction does not require the terminal 34 residues.

**Figure 5:**
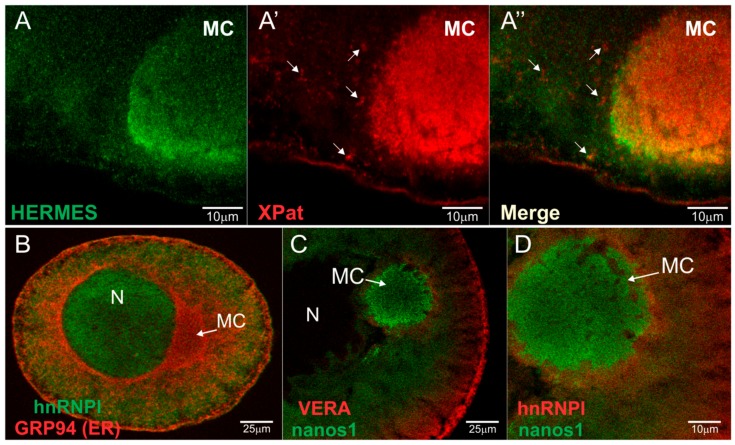
Cellular distribution of Hermes/Rbpms, hnRNP I, Vg1RBP/Vera, and Xpat proteins in the stage I/II oocytes. Confocal images showing immunofluorescence (IF) of stage I/II oocyte with (**A**) anti-Hermes/Rbpms (green) and (**A’**) anti-Xpat (red) antibodies; (**A’’**) Superimposed image. White arrows indicate Xpat and Hermes/Rbpms particles are not identical; (**B**) Superimposed images of hnRNP I (**green**) and GRP94 (**red**) proteins. Co-staining with anti-GRP94 reveals the ER enriched within the MC, cortex, and perinuclear region. Note that hnRNP I is excluded from the MC but is present in the nucleus and cytoplasm; (**C**) Merged images of endogenous Vg1RBP/Vera (**red**); or **(D)** hnRNP I (**red**) with Alexa 488-labeled *nanos* 3′UTR injected 20 h before fixation (**green**). Note that while *nanos* RNA localizes to the MC, both Vg1RB/Vera and hnRNP I are excluded from it. N: nucleus; MC: mitochondrial cloud. Scale bars are as indicated.

Xpat protein is a major structural component of the germ plasm and is thought to provide a scaffold for other germ plasm components [58]. To characterize the relationship between endogenous Hermes/Rbpms and Xpat within the MC, stage I/II oocytes were fix and stained with the corresponding antibodies followed by confocal microscopy. Dissimilar patterns were observed for the two proteins. Hermes/Rbpms appeared as fine particles, smaller and less distinct than those for Xpat, but falling within the larger defined Xpat structure. The Confocal images are consistent with the idea that Hermes/Rbpms is directly associated with localized RNAs whereas Xpat has more of a global role in germ plasm structure within which RNP particles are embedded, including germinal granules (Figure 5A,A’). Our results are consistent with those of Nijjar and Woodland [53,59]. Next we examined the two endogenous late pathway *Vg1* RNA binding proteins Vg1RBP/Vera and hnRNP I in relation to labeled injected early pathway *nanos* RNA. hnRNP I was found in the nucleus and appeared to partially co-localize with RNA just outside the MC (Figure 5B,D). Both Vg1RBP/Vera and hnRNP I however, are excluded from the MC suggesting the RNP particles destined for the germ plasm are different from those following the late *Vg1* localization pathway (Figure 5C,D).

We attempted to visually capture Hermes/Rbpms-*nanos* RNA interaction within the ooplasm during the process of RNA localization into the MC. Fluorescently tagged *nanos* 3′UTR RNA was injected into stage I oocytes, and after 5 or 20 h, oocytes were fixed. The 3′UTR of nanos RNA was used in order to eliminate the possibility of *nanos* translation, an event that could obscure RNA-protein interactions. Endogenous Hermes/Rbpms protein was identified by immunofluorescence after staining with an anti-Hermes/Rbpms antibody. Regardless of conditions or time, we could not detect any co-localization between injected *nanos* 3′UTR RNA and endogenous Hermes/Rbpms (data not shown). Thus, we were unable to use this approach to extend our analysis *in vivo*.

### 3.5. Hermes/Rbpms Associates with Nanos RNA within the Nucleus of Stage I Oocytes

**Figure 6:**
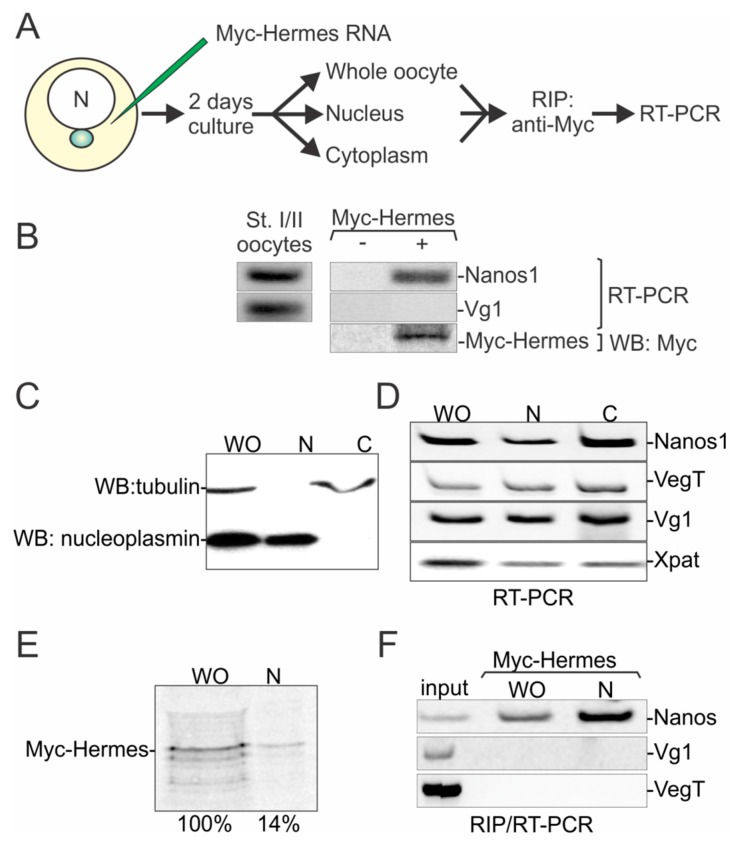
Hermes/Rbpms associates with *nanos* but not *Vg1* RNA in the nucleus. (**A**) Overall experimental design. Myc-Hermes/Rbpms mRNA was injected into stage I/II oocytes. After culturing for two days, either myc-Hermes/Rbpms protein was immunoprecipitated (IP) with anti-myc antibody from whole oocytes only or the nuclear (N) and cytoplasmic (C) fractions were manually isolated before IP with anti-myc antibody. In either design, RNA was extracted from the resulting pellet and analyzed with specific primers for *Vg1* or *nanos* RNA by RT-PCR; (**B**) Myc-Hermes/Rbpms interacts with *nanos* but not *Vg1* RNA *in vivo*. Control RT-PCR with whole oocytes shows that *nanos* and *Vg1* RNAs were present. Control western blot shows that myc-Hermes/Rbpms was translated after injection; (**C**) The nuclear and cytoplasmic fractions were manually isolated from stage I/II oocytes. Tubulin (cytoplasmic marker) and nucleoplasmin (nuclear marker) were detected by western blot analysis and show clean separation; (**D**) Late and Early Pathway RNAs accumulate in nucleus concurrently. RNAs were analyzed by RT-PCR using specific primers; (**E**) Western blot analysis showed newly translated Hermes/Rbpms in the nucleus; (**F**) Hermes/Rbpms interacts with *nanos* RNA in the nucleus, but not *Vg1* or *VegT*. WO: whole oocyte; Uninjected oocytes served as negative controls. Blue sphere (MC); N (nucleus).

In our assay, *nanos* RNA associated with Hermes/Rbpms while the late pathway RNA *Vg1* did not. These results suggested that Hermes/Rbpms could discriminate between endogenous RNAs that employ different localization pathways. To determine if such discrimination occurs *in vivo*, myc-Hermes/Rbpms RNA was injected into stage I oocytes. The oocytes were cultured for two days to allow translation to occur (Figure 6A). The oocytes were then homogenized, and myc-antibody was used to immunoprecipitate (IP) the newly translated myc-Hermes/Rbpms from the supernatant. RNA was isolated from the IP pellet and the presence of *nanos* and *Vg1* RNAs determined by RT-PCR using specific primers. While *nanos* RNA was recovered in the precipitate, *Vg1* RNA was not detected (Figure 6B). From these results, we conclude that *nanos* RNA associates with Hermes/Rbpms *in vivo* in stage I oocytes.

As shown in Figure 1, some of the endogenous Hermes/Rbpms protein is nuclear, raising the possibility that Hermes/Rbpms associates with *nanos* RNA there during the earliest steps of RNA localization. We first determined if *nanos*, and two late pathway RNAs, *Vg1* and *VegT*, were present concurrently in stage I nuclei. Nuclei were manually isolated together with the enucleated cytoplasm. Western blotting with antibodies against tubulin and nucleoplasmin were used to assess the level of contamination between the isolated cytoplasm and nuclear samples respectively (Figure 6C). *VegT*, *Vg1* and *nanos* RNA were all shown to be present within nuclei of stage I oocytes and with similar nuclear to cytoplasmic ratios (Figure 6D). To determine if Hermes/Rbpms could discriminate between these three RNAs, Myc-*hermes/rbpms* RNA was injected into isolated stage I oocytes and oocytes cultured for two days. Cleared supernatants were prepared from whole and nuclear samples and Myc-Hermes/Rbpms immunoprecipitated from them. As expected, newly synthesized Myc-Hermes/Rbpms protein was found in the nucleus as well as the cytoplasm at the end of the two-day culture period (Figure 6E). RNA was isolated from the IP pellet and the presence of *nanos*, *Vg1* and *VegT* RNAs determined by RT-PCR using specific primers. While *nanos* RNA was recovered in the precipitate from the whole and nuclear fractions, *Vg1* and *VegT* RNA were not detected (Figure 6F). Taken together, our results show that interaction between Hermes/Rbpms and *nanos* RNA initiates within the nucleus. Further, this interaction is specific as *Vg1* and *VegT* RNAs are not recognized.

## 4. Discussion

Germline development requires proper assembly of specific RNAs and proteins within the germ plasm region of the MC. At the same time, RNAs encoding proteins with exclusive roles in somatic fates must be excluded. The consequence of mis-expression of somatic determinants within the germline is sterility. However, the mechanism(s) for sorting RNAs into their correct locations remains poorly understood. Early recognition by RNA-binding proteins within the nucleus is one attractive mechanism for initiating an essential sorting step in RNA localization. In this report, we show that Hermes/Rbpms recognizes *nanos*, an essential germline component, but not *Vg1* or *VegT* RNA, within the nucleus. Four lines of evidence support a role for Hermes/Rbpms as a key protein component of the *nanos* RNP particle: (1) Hermes/Rbpms co-localizes with *nanos* RNA within the MC and is a component of germinal granules ([25], Figure 1); (2) Hermes/Rbpms binds *nanos* but not *Vg1* RNA in both *in vitro* and *in vivo* assays (Figure 2 and Figure 6); (3) The UGCAC repeats that are essential for *nanos* localization to the MC germ plasm are also required for Hermes/Rbpms binding to the MCLS (Figure 2B); (4) The C-terminal 34 residues of Hermes/Rbpms are required for Hermes/Rbpms to form homodimers. Deletion of these residues, even in the presence of the Hermes/Rbpms RRM, prevents Hermes/Rbpms from binding to *nanos*.

Studies on *Vg1* RNA have provided support that the late RNA localization pathway initiates in the nucleus [42,60]. Those studies show that Vg1RBP/Vera and hnRNP I directly contact each other and bind *Vg1* RNA in the nucleus. Interestingly, once the *Vg1* RNP exits the nucleus and enters the cytoplasm, additional proteins are now added and Vg1RBP/Vera and hnRNP I are no longer in direct contact. Staufen is one of the additional proteins found in the cytoplasmic Vg1RNP complex. Staufen likely initiates association with the motor protein kinesin and thus promotes active transport to the vegetal pole [21,42]. Therefore, a dynamic remodeling of the RNP complex takes place that could account for *Vg1* active transport to the vegetal cortex using the late pathway.

There are parallels to be drawn between the *Vg1* studies and those reported here. *Nanos* RNA passes through five cellular compartments before localizing into germinal granules: the nucleus, cytoplasm, MC, ER, and germ plasm. None of these steps require active transport. Each cellular location could involve remodeling, that is proteins added, subtracted, or modified. Hermes/Rbpms association with *nanos* RNA, but not with *Vg1* or *VegT* RNAs within the nucleus suggests that Hermes/Rbpms binding might initiate a sorting pathway that terminates with *nanos*/Hermes/Rbpms within the germinal granules.

In the assay shown in Figure 2, *nanos* pulled down Hermes/Rbpms only when the reticulocyte lysate was supplemented with a protein extract from stage I oocytes. These results strongly suggested other proteins were required for Hermes/Rbpms binding to the *nanos* 3′UTR [48]. Could hnRNP I be part of an early nuclear *nanos* RNP? *In vitro* UV-crosslinking experiments show a specific interaction between hnRNP I and *nanos* RNA, however an association *in vivo* remains unclear [6]. Interestingly, both Vg1RBP/Vera and hnRNP I are excluded from the MC, so any association with *nanos* would be temporary (Figure 5). hnRNP I does interact directly with Hermes/Rbpms protein absent any RNA as revealed in the yeast-2-hybrid assay (Figure 4). Hermes/Rbpms is also found throughout the oocyte and is known to interact with other RNAs [52]. Further studies are required to establish if indeed hnRNP I is part of the endogenous Hermes/Rbpms/*nanos* RNP particle.

Nijjar and Woodland [53] have recently identified four other proteins that directly interact with Hermes/Rbpms: Xvelo splice variant (SV), Xvelo-full length (FL) and RNA-binding proteins Rbm42b and Rbm24b. They used a bimolecular fluorescence complementation (BiFC) approach to show likely protein-protein interactions after injection of tagged candidates. However, their assays were done in fully-grown oocytes (stage VI) and not at stages when *nanos* normally localizes and a MC is present. The protein components of the *nanos* RNP will likely change depending on the oocyte stage examined and localization mechanism employed. Indeed, tagged-*nanos* RNA injected into late stage oocytes is capable of using active transport to the vegetal pole but also has been shown to enter germ plasm structures by diffusion and entrapment at these late stages [59]. Although these interactions and subcellular locations are based on over-expression of reporters in the ooplasm, they do indicate what is possible [59]. With these caveats in mind, their findings suggest that Hermes/Rbpms likely associates with Rbm42b in the nucleus. Rbm42b also co-localizes with *nanos* RNA in late stage oocytes. From our proteomic analysis of the MC, Rbm42b is present (unpublished data) and could be a good candidate for one of the factors required for Hermes/Rbpms *nanos* RNA binding. Their analysis further suggests that Rbm24b and XveloSV associate with each other in the cytoplasm, but not in the nucleus indicating dynamic remodeling likely does occur.

Hermes/Rbpms colocalization with Xvelo within the germ plasm particles is a significant finding [53]. Xvelo is the homologue to Bucky ball in zebrafish, a gene required for the formation of the MC (Balbiani body) and oocyte polarity. By an unknown mechanism, Bucky ball is also linked to the germline as its over-expression results in more PGCs being formed [61]. Endogenous VeloFL does not appear to be nuclear but is cytoplasmic and enriched within the MC [53]. Thus Xvelo may join the Hermes/Rbpms/*nanos* RNP after it exits the nucleus.

The question remains as to what function Hermes/Rbpms performs within the germ plasm. Hermes/Rbpms has been studied in a wide range of cell types and tissues [62,63,64]. A conserved aspect is Hermes/Rbpms ability to oligomerize with itself and other proteins to form RNP granules. Within these RNP granules, Hermes/Rbpms is associated with repression whether in retinal ganglion cells (RGCs) [62,63], *Xenopus* germinal granules [49,65] or mouse and human cell lines [64]. Hermes/Rbpms appears to negatively regulate RINGO/Spy and Mos, RNAs involved in meiotic maturation and early cleavage respectively [49]. Hermes/Rbpms was shown to repress activator protein-1 (AP-1) signaling through cFos, cJun, and Smad3, a pathway implicated in tumor growth and progression [64]. Hermes/Rbpms blocks the interaction of these complexes and their recruitment to the promoter regions of AP-1 target genes. Similarly, over-expression of Hermes/Rbpms causes significant reduction in NKx2-5 expression, a transcription factor required for heart development [48].

In RGCs, Hermes/Rbpms forms RNP granules by interacting with other RNA binding proteins NonO, PSF, and G3BP1. Proteonomic analysis of the *Xenopus* MC revealed the presence of NonO (our unpublished results). Hermes/Rbpms containing granules are transported down growing axons. Hermes/Rbpms gain-of-function and loss-of function experiments *in vivo* in RGCs reveal a complex phenotype consistent with a role in balancing synaptogenesis and axon arborization [62]. In that study, a dominant negative form of Hermes/Rbpms was constructed lacking the C-terminus. *In vivo*, this mutant failed to form granules and localize properly. Their results are consistent with our findings that the C-terminus is required for Hermes/Rbpms to oligomerize and to bind *nanos* RNA.

In our studies, Hermes/Rbpms was also shown to form RNP particles within the nucleus, cytoplasm and MC. Once in the germ plasm, Hermes/Rbpms forms germinal granules that contain *nanos* RNA (Figure 1) [49]. Although *nanos* is transcribed very early in oogenesis, it is not translated at any time during oogenesis, but only after fertilization [13,65]. Premature translation in oocytes results in abnormal development [66]. Therefore, as soon as *nanos* RNA is transcribed, it must be translationally silenced before its incorporation into germinal granules. Once within germinal granules, *nanos* is stable for many months.

## 5. Conclusions

Our data are consistent with a model where multiple copies of Hermes/Rbpms specifically bind *nanos* RNA in the nucleus of stage I oocytes. Hermes/Rbpms may require a co-factor to bind RNA efficiently as it contains only one RRM, perhaps not sufficient by itself to bind RNA (Figure 2; [48]). Nuclear Hermes/Rbpms recruits other proteins to the *nanos* RNP particle and these may be Rbm42b and XveloFL. The Hermes/Rbpms complex does not bind *Vg1* or *VegT* RNA. Upon exiting the nucleus, these proteins prevent *nanos* from being translated. The *nanos* RNP particle diffuses through the cytoplasm and is trapped on an ER component (Xpat?) within the MC [6,58]. Within the MC, other RNA binding proteins likely associate, but in the final germinal granule, Hermes/Rbpms is the only protein identified to date. Dynamic remodeling of the *nanos* RNP particle as it moves to the germ plasm and is incorporated within germinal granules is an important concept. Additional studies will be required to clarify the endogenous *nanos* RNP particle during its different localization steps that culminate in the germinal granule.
